# Supplementary material for: Meta-Analysis and Gene Set Enrichment Relative to ER Status Reveal Elevated Activity of MYC and E2F in the “Basal” Breast Cancer Subgroup
Source: PLoS One. 2009 Mar 9;4(3):e4710. doi: 10.1371/journal.pone.0004710 (PMC2650420; doi:10.1371/journal.pone.0004710)
Supplement: Dataset S1 — GSEA results from first screen. The file contains a zipped archive of the HTML results of the second GSEA screen. Reports for enriched or depleted datasets may be accessed from the “index.html” link within the my_analysis.GseaPreranked.1219975950625.rpt.zip ZIP archive. Enrichment plots are available for each of the 50 most enriched and depleted gene sets. (13.32 MB ZIP) [file pone.0004710.s001.zip › my_analysis.GseaPreranked.1219975950625.rpt.zip!//BRCA_ER_POS.html]

Details for gene set BRCA\_ER\_POS[GSEA]

|  || Dataset | zSco.ERneg.to.ERpos\_collapsed |
| Phenotype | NoPhenotypeAvailable |
| Upregulated in class | na\_neg |
| GeneSet | BRCA\_ER\_POS |
| Enrichment Score (ES) | -0.7881392 |
| Normalized Enrichment Score (NES) | -4.396279 |
| Nominal p-value | 0.0 |
| FDR q-value | 0.0 |
| FWER p-Value | 0.0 |
Table: GSEA Results Summary

  

Fig 1: Enrichment plot: BRCA\_ER\_POS      
 Profile of the Running ES Score & Positions of GeneSet Members on the Rank Ordered List

  

| PROBE | GENE SYMBOL | GENE\_TITLE | RANK IN GENE LIST | RANK METRIC SCORE | RUNNING ES | CORE ENRICHMENT || 1 | CD47 |  |  | 1412 | 3.030 | -0.1079 | No |
| 2 | KIAA0195 |  |  | 2461 | 2.219 | -0.1879 | No |
| 3 | SYNGR1 |  |  | 2512 | 2.194 | -0.1903 | No |
| 4 | USP22 |  |  | 2924 | 1.973 | -0.2210 | No |
| 5 | ABCA2 |  |  | 3367 | 1.764 | -0.2542 | No |
| 6 | PTPN1 |  |  | 4004 | 1.511 | -0.3026 | No |
| 7 | ITPK1 |  |  | 4264 | 1.408 | -0.3218 | No |
| 8 | DNAJA4 |  |  | 4395 | 1.357 | -0.3310 | No |
| 9 | CROCC |  |  | 4518 | 1.312 | -0.3396 | No |
| 10 | FLJ33790 |  |  | 4530 | 1.308 | -0.3396 | No |
| 11 | FGF18 |  |  | 4851 | 1.206 | -0.3637 | No |
| 12 | KIAA0515 |  |  | 5110 | 1.113 | -0.3830 | No |
| 13 | EML2 |  |  | 5249 | 1.064 | -0.3930 | No |
| 14 | LANCL1 |  |  | 5254 | 1.062 | -0.3926 | No |
| 15 | AGPAT7 |  |  | 5609 | 0.942 | -0.4195 | No |
| 16 | PCSK6 |  |  | 5798 | 0.878 | -0.4335 | No |
| 17 | PPP2R5A |  |  | 6008 | 0.810 | -0.4492 | No |
| 18 | BRD8 |  |  | 6010 | 0.808 | -0.4488 | No |
| 19 | BLVRA |  |  | 6052 | 0.794 | -0.4514 | No |
| 20 | RPS14 |  |  | 6165 | 0.761 | -0.4596 | No |
| 21 | GREB1 |  |  | 6264 | 0.727 | -0.4667 | No |
| 22 | ALDH6A1 |  |  | 6317 | 0.711 | -0.4703 | No |
| 23 | HDGFRP3 |  |  | 6446 | 0.668 | -0.4798 | No |
| 24 | CDS1 |  |  | 6529 | 0.639 | -0.4857 | No |
| 25 | H2AFV |  |  | 6569 | 0.627 | -0.4883 | No |
| 26 | ZBTB40 |  |  | 6761 | 0.568 | -0.5028 | No |
| 27 | HIST1H2AK |  |  | 6856 | 0.533 | -0.5098 | No |
| 28 | PTGER3 |  |  | 6899 | 0.519 | -0.5127 | No |
| 29 | SNX1 |  |  | 6944 | 0.505 | -0.5158 | No |
| 30 | RPLP1 |  |  | 6999 | 0.488 | -0.5196 | No |
| 31 | PATZ1 |  |  | 7233 | 0.409 | -0.5375 | No |
| 32 | DHRS7 |  |  | 7249 | 0.401 | -0.5384 | No |
| 33 | RAB11A |  |  | 7295 | 0.384 | -0.5416 | No |
| 34 | PRKAR2B |  |  | 7314 | 0.378 | -0.5428 | No |
| 35 | ASAH1 |  |  | 7329 | 0.372 | -0.5436 | No |
| 36 | SPDEF |  |  | 7330 | 0.372 | -0.5433 | No |
| 37 | MUC1 |  |  | 7332 | 0.371 | -0.5432 | No |
| 38 | CELSR1 |  |  | 7436 | 0.332 | -0.5510 | No |
| 39 | RMND5B |  |  | 7446 | 0.330 | -0.5514 | No |
| 40 | ASTN2 |  |  | 7528 | 0.296 | -0.5575 | No |
| 41 | CTBP2 |  |  | 7675 | 0.239 | -0.5687 | No |
| 42 | VPS13B |  |  | 7699 | 0.231 | -0.5704 | No |
| 43 | ZHX3 |  |  | 7714 | 0.226 | -0.5713 | No |
| 44 | AFF1 |  |  | 7753 | 0.212 | -0.5741 | No |
| 45 | KIAA0241 |  |  | 7759 | 0.208 | -0.5744 | No |
| 46 | SURF1 |  |  | 7829 | 0.178 | -0.5796 | No |
| 47 | CFB |  |  | 7854 | 0.165 | -0.5814 | No |
| 48 | LRRC49 |  |  | 7891 | 0.150 | -0.5841 | No |
| 49 | APRIN |  |  | 7954 | 0.121 | -0.5888 | No |
| 50 | INPP4B |  |  | 8044 | 0.088 | -0.5957 | No |
| 51 | MPDZ |  |  | 8133 | 0.062 | -0.6025 | No |
| 52 | SLC9A5 |  |  | 8303 | 0.000 | -0.6157 | No |
| 53 | NCDN |  |  | 8370 | -0.025 | -0.6208 | No |
| 54 | GOLGA1 |  |  | 8409 | -0.042 | -0.6237 | No |
| 55 | PCM1 |  |  | 8453 | -0.057 | -0.6270 | No |
| 56 | MSX2 |  |  | 8598 | -0.128 | -0.6381 | No |
| 57 | PCDH1 |  |  | 8699 | -0.167 | -0.6458 | No |
| 58 | ATPAF2 |  |  | 8726 | -0.180 | -0.6477 | No |
| 59 | UNC13B |  |  | 8808 | -0.212 | -0.6539 | No |
| 60 | C8ORF4 |  |  | 8860 | -0.237 | -0.6577 | No |
| 61 | BTBD9 |  |  | 8953 | -0.290 | -0.6646 | No |
| 62 | KRT8 |  |  | 8956 | -0.291 | -0.6646 | No |
| 63 | NEBL |  |  | 8997 | -0.310 | -0.6675 | No |
| 64 | WNT5A |  |  | 9278 | -0.447 | -0.6890 | No |
| 65 | KRT8L2 |  |  | 9305 | -0.458 | -0.6907 | No |
| 66 | LIPE |  |  | 9322 | -0.469 | -0.6916 | No |
| 67 | KIAA0100 |  |  | 9323 | -0.470 | -0.6913 | No |
| 68 | TUBA2 |  |  | 9463 | -0.545 | -0.7017 | No |
| 69 | NEO1 |  |  | 9529 | -0.577 | -0.7064 | No |
| 70 | WFDC2 |  |  | 9530 | -0.577 | -0.7060 | No |
| 71 | ABCC8 |  |  | 9579 | -0.607 | -0.7093 | No |
| 72 | BCL2 |  |  | 9582 | -0.608 | -0.7091 | No |
| 73 | PRKCBP1 |  |  | 9596 | -0.615 | -0.7097 | No |
| 74 | KIAA1018 |  |  | 9648 | -0.645 | -0.7132 | No |
| 75 | LOC220594 |  |  | 9739 | -0.691 | -0.7197 | No |
| 76 | BBC3 |  |  | 9752 | -0.695 | -0.7202 | No |
| 77 | KIAA0888 |  |  | 9760 | -0.701 | -0.7202 | No |
| 78 | ZNF219 |  |  | 9805 | -0.727 | -0.7232 | No |
| 79 | ELOVL5 |  |  | 9826 | -0.741 | -0.7242 | No |
| 80 | SH2B1 |  |  | 9838 | -0.747 | -0.7246 | No |
| 81 | SLC4A7 |  |  | 9843 | -0.751 | -0.7243 | No |
| 82 | KIAA1467 |  |  | 9847 | -0.752 | -0.7241 | No |
| 83 | ITPR1 |  |  | 10079 | -0.894 | -0.7414 | No |
| 84 | IGF2 |  |  | 10097 | -0.906 | -0.7421 | No |
| 85 | CACNG1 |  |  | 10225 | -0.973 | -0.7514 | No |
| 86 | ICA1 |  |  | 10287 | -1.020 | -0.7554 | No |
| 87 | ESR1 |  |  | 10292 | -1.026 | -0.7550 | No |
| 88 | GDF15 |  |  | 10318 | -1.041 | -0.7562 | No |
| 89 | MPI |  |  | 10447 | -1.118 | -0.7654 | No |
| 90 | DEAF1 |  |  | 10455 | -1.127 | -0.7652 | No |
| 91 | HSPA1A |  |  | 10471 | -1.135 | -0.7656 | No |
| 92 | SYT13 |  |  | 10474 | -1.136 | -0.7650 | No |
| 93 | CRIM1 |  |  | 10502 | -1.163 | -0.7663 | No |
| 94 | ARMCX2 |  |  | 10512 | -1.173 | -0.7662 | No |
| 95 | CHRD |  |  | 10562 | -1.216 | -0.7691 | No |
| 96 | LIN7A |  |  | 10563 | -1.216 | -0.7683 | No |
| 97 | SLC43A1 |  |  | 10570 | -1.220 | -0.7679 | No |
| 98 | FRZB |  |  | 10606 | -1.249 | -0.7698 | No |
| 99 | HNRPA1 |  |  | 10609 | -1.250 | -0.7691 | No |
| 100 | NUDT2 |  |  | 10651 | -1.280 | -0.7714 | No |
| 101 | RUTBC1 |  |  | 10696 | -1.314 | -0.7739 | No |
| 102 | CDH1 |  |  | 10717 | -1.328 | -0.7746 | No |
| 103 | NPY1R |  |  | 10744 | -1.349 | -0.7756 | No |
| 104 | CNKSR1 |  |  | 10749 | -1.352 | -0.7750 | No |
| 105 | ZBTB16 |  |  | 10803 | -1.389 | -0.7782 | No |
| 106 | EPHX2 |  |  | 10808 | -1.391 | -0.7776 | No |
| 107 | POLB |  |  | 10862 | -1.435 | -0.7807 | No |
| 108 | NEK9 |  |  | 10871 | -1.443 | -0.7803 | No |
| 109 | ARL3 |  |  | 10877 | -1.447 | -0.7797 | No |
| 110 | HEXIM1 |  |  | 10897 | -1.472 | -0.7802 | No |
| 111 | GOLPH3L |  |  | 10909 | -1.486 | -0.7800 | No |
| 112 | C18ORF1 |  |  | 10918 | -1.494 | -0.7796 | No |
| 113 | DLG3 |  |  | 10923 | -1.502 | -0.7789 | No |
| 114 | AGTR1 |  |  | 10928 | -1.504 | -0.7782 | No |
| 115 | BAG1 |  |  | 10930 | -1.508 | -0.7772 | No |
| 116 | DET1 |  |  | 10952 | -1.531 | -0.7778 | No |
| 117 | PRKAR1A |  |  | 10989 | -1.556 | -0.7795 | No |
| 118 | PER2 |  |  | 11054 | -1.618 | -0.7834 | No |
| 119 | CITED1 |  |  | 11109 | -1.670 | -0.7864 | No |
| 120 | LOC400451 |  |  | 11111 | -1.673 | -0.7854 | No |
| 121 | MID1IP1 |  |  | 11122 | -1.682 | -0.7850 | No |
| 122 | IMPACT |  |  | 11142 | -1.695 | -0.7853 | No |
| 123 | BAG3 |  |  | 11166 | -1.716 | -0.7859 | No |
| 124 | DOK1 |  |  | 11182 | -1.731 | -0.7859 | No |
| 125 | DIP2C |  |  | 11206 | -1.747 | -0.7865 | No |
| 126 | KIAA0802 |  |  | 11209 | -1.748 | -0.7854 | No |
| 127 | NEDD4L |  |  | 11210 | -1.749 | -0.7842 | No |
| 128 | PHF15 |  |  | 11215 | -1.755 | -0.7833 | No |
| 129 | FBXL2 |  |  | 11222 | -1.760 | -0.7826 | No |
| 130 | CLCN3 |  |  | 11233 | -1.771 | -0.7821 | No |
| 131 | GLA |  |  | 11269 | -1.804 | -0.7836 | No |
| 132 | LOC51149 |  |  | 11283 | -1.812 | -0.7834 | No |
| 133 | CLGN |  |  | 11294 | -1.818 | -0.7829 | No |
| 134 | ARNT2 |  |  | 11302 | -1.826 | -0.7822 | No |
| 135 | OVOL2 |  |  | 11341 | -1.872 | -0.7839 | No |
| 136 | RSAD1 |  |  | 11397 | -1.930 | -0.7868 | Yes |
| 137 | FLJ10916 |  |  | 11415 | -1.947 | -0.7868 | Yes |
| 138 | CYP2B6 |  |  | 11429 | -1.967 | -0.7865 | Yes |
| 139 | STXBP1 |  |  | 11435 | -1.972 | -0.7855 | Yes |
| 140 | NT5C |  |  | 11443 | -1.983 | -0.7847 | Yes |
| 141 | FER1L3 |  |  | 11462 | -2.000 | -0.7847 | Yes |
| 142 | FBXO3 |  |  | 11469 | -2.004 | -0.7838 | Yes |
| 143 | CRELD1 |  |  | 11471 | -2.006 | -0.7825 | Yes |
| 144 | KIAA0649 |  |  | 11480 | -2.011 | -0.7817 | Yes |
| 145 | CREBL2 |  |  | 11536 | -2.074 | -0.7846 | Yes |
| 146 | C14ORF132 |  |  | 11544 | -2.083 | -0.7837 | Yes |
| 147 | DBNDD2 |  |  | 11547 | -2.085 | -0.7824 | Yes |
| 148 | CRAT |  |  | 11548 | -2.086 | -0.7810 | Yes |
| 149 | WDR6 |  |  | 11563 | -2.098 | -0.7806 | Yes |
| 150 | C9ORF46 |  |  | 11599 | -2.123 | -0.7819 | Yes |
| 151 | PISD |  |  | 11602 | -2.127 | -0.7806 | Yes |
| 152 | KCNJ3 |  |  | 11608 | -2.132 | -0.7795 | Yes |
| 153 | PLK2 |  |  | 11632 | -2.151 | -0.7798 | Yes |
| 154 | GLRB |  |  | 11635 | -2.152 | -0.7785 | Yes |
| 155 | CYB5A |  |  | 11639 | -2.157 | -0.7772 | Yes |
| 156 | IL13RA1 |  |  | 11641 | -2.160 | -0.7758 | Yes |
| 157 | MORF4L2 |  |  | 11645 | -2.163 | -0.7745 | Yes |
| 158 | NOVA1 |  |  | 11652 | -2.168 | -0.7735 | Yes |
| 159 | STC2 |  |  | 11658 | -2.176 | -0.7724 | Yes |
| 160 | LASP1 |  |  | 11661 | -2.179 | -0.7711 | Yes |
| 161 | GADD45G |  |  | 11665 | -2.186 | -0.7698 | Yes |
| 162 | RICS |  |  | 11667 | -2.191 | -0.7684 | Yes |
| 163 | C4ORF16 |  |  | 11669 | -2.191 | -0.7669 | Yes |
| 164 | RAMP1 |  |  | 11680 | -2.202 | -0.7662 | Yes |
| 165 | TRAK1 |  |  | 11684 | -2.205 | -0.7649 | Yes |
| 166 | AQR |  |  | 11705 | -2.226 | -0.7649 | Yes |
| 167 | SLC1A1 |  |  | 11706 | -2.228 | -0.7634 | Yes |
| 168 | ISOC1 |  |  | 11714 | -2.233 | -0.7624 | Yes |
| 169 | TRAK2 |  |  | 11726 | -2.247 | -0.7617 | Yes |
| 170 | TSPAN15 |  |  | 11730 | -2.249 | -0.7604 | Yes |
| 171 | CCNI |  |  | 11736 | -2.255 | -0.7592 | Yes |
| 172 | BLCAP |  |  | 11742 | -2.259 | -0.7581 | Yes |
| 173 | GAS8 |  |  | 11744 | -2.261 | -0.7566 | Yes |
| 174 | QSCN6 |  |  | 11754 | -2.269 | -0.7557 | Yes |
| 175 | VEZF1 |  |  | 11771 | -2.295 | -0.7554 | Yes |
| 176 | SOX13 |  |  | 11786 | -2.313 | -0.7549 | Yes |
| 177 | ABCG1 |  |  | 11792 | -2.319 | -0.7537 | Yes |
| 178 | JMJD2B |  |  | 11820 | -2.348 | -0.7542 | Yes |
| 179 | SKAP1 |  |  | 11833 | -2.353 | -0.7535 | Yes |
| 180 | CTR9 |  |  | 11840 | -2.360 | -0.7523 | Yes |
| 181 | SLC30A1 |  |  | 11841 | -2.361 | -0.7507 | Yes |
| 182 | AHNAK |  |  | 11844 | -2.364 | -0.7492 | Yes |
| 183 | LRP2 |  |  | 11846 | -2.366 | -0.7477 | Yes |
| 184 | SCAMP1 |  |  | 11847 | -2.367 | -0.7460 | Yes |
| 185 | CRI1 |  |  | 11851 | -2.370 | -0.7446 | Yes |
| 186 | SLC16A5 |  |  | 11867 | -2.396 | -0.7442 | Yes |
| 187 | ZDHHC17 |  |  | 11868 | -2.399 | -0.7425 | Yes |
| 188 | RTN1 |  |  | 11872 | -2.403 | -0.7411 | Yes |
| 189 | GLUL |  |  | 11874 | -2.404 | -0.7395 | Yes |
| 190 | GJA1 |  |  | 11883 | -2.421 | -0.7385 | Yes |
| 191 | SRP14 |  |  | 11898 | -2.437 | -0.7379 | Yes |
| 192 | NGRN |  |  | 11903 | -2.440 | -0.7365 | Yes |
| 193 | RHOBTB2 |  |  | 11904 | -2.440 | -0.7348 | Yes |
| 194 | SOX12 |  |  | 11906 | -2.441 | -0.7332 | Yes |
| 195 | TOM1L1 |  |  | 11945 | -2.492 | -0.7345 | Yes |
| 196 | UNG2 |  |  | 11947 | -2.493 | -0.7328 | Yes |
| 197 | GPRC5C |  |  | 11948 | -2.493 | -0.7311 | Yes |
| 198 | HD |  |  | 11959 | -2.513 | -0.7302 | Yes |
| 199 | GPC1 |  |  | 11965 | -2.518 | -0.7288 | Yes |
| 200 | BTD |  |  | 11967 | -2.521 | -0.7272 | Yes |
| 201 | SHC2 |  |  | 11968 | -2.522 | -0.7254 | Yes |
| 202 | GPHN |  |  | 11980 | -2.540 | -0.7245 | Yes |
| 203 | SPOP |  |  | 11984 | -2.544 | -0.7230 | Yes |
| 204 | RARA |  |  | 11993 | -2.552 | -0.7219 | Yes |
| 205 | MAP3K1 |  |  | 11996 | -2.557 | -0.7203 | Yes |
| 206 | PDE6B |  |  | 12010 | -2.573 | -0.7195 | Yes |
| 207 | SSBP2 |  |  | 12052 | -2.644 | -0.7209 | Yes |
| 208 | ERBB4 |  |  | 12058 | -2.650 | -0.7194 | Yes |
| 209 | BTF3 |  |  | 12059 | -2.650 | -0.7176 | Yes |
| 210 | THRAP2 |  |  | 12063 | -2.652 | -0.7160 | Yes |
| 211 | ARHGEF16 |  |  | 12064 | -2.653 | -0.7142 | Yes |
| 212 | SERPINA5 |  |  | 12084 | -2.680 | -0.7138 | Yes |
| 213 | C20ORF28 |  |  | 12090 | -2.693 | -0.7124 | Yes |
| 214 | GDI1 |  |  | 12108 | -2.714 | -0.7118 | Yes |
| 215 | DDHD2 |  |  | 12113 | -2.720 | -0.7103 | Yes |
| 216 | PTP4A2 |  |  | 12117 | -2.722 | -0.7086 | Yes |
| 217 | GOLPH2 |  |  | 12127 | -2.743 | -0.7074 | Yes |
| 218 | TP53AP1 |  |  | 12136 | -2.753 | -0.7062 | Yes |
| 219 | CELSR2 |  |  | 12140 | -2.759 | -0.7045 | Yes |
| 220 | KIAA1324 |  |  | 12142 | -2.762 | -0.7027 | Yes |
| 221 | TRAPPC6A |  |  | 12148 | -2.765 | -0.7011 | Yes |
| 222 | EVL |  |  | 12166 | -2.798 | -0.7005 | Yes |
| 223 | MYLIP |  |  | 12172 | -2.805 | -0.6990 | Yes |
| 224 | KIAA0141 |  |  | 12187 | -2.824 | -0.6981 | Yes |
| 225 | POLR2L |  |  | 12192 | -2.836 | -0.6965 | Yes |
| 226 | QDPR |  |  | 12196 | -2.838 | -0.6948 | Yes |
| 227 | SERPINA6 |  |  | 12203 | -2.846 | -0.6933 | Yes |
| 228 | PVALB |  |  | 12208 | -2.849 | -0.6916 | Yes |
| 229 | PTPN13 |  |  | 12213 | -2.856 | -0.6900 | Yes |
| 230 | SEMA3G |  |  | 12221 | -2.872 | -0.6886 | Yes |
| 231 | KIF13B |  |  | 12226 | -2.881 | -0.6869 | Yes |
| 232 | DHRS2 |  |  | 12228 | -2.884 | -0.6850 | Yes |
| 233 | CHAD |  |  | 12230 | -2.889 | -0.6831 | Yes |
| 234 | RETSAT |  |  | 12237 | -2.897 | -0.6815 | Yes |
| 235 | SLC22A18 |  |  | 12251 | -2.912 | -0.6805 | Yes |
| 236 | PIGG |  |  | 12252 | -2.912 | -0.6785 | Yes |
| 237 | LRIG1 |  |  | 12253 | -2.914 | -0.6765 | Yes |
| 238 | PDE8B |  |  | 12262 | -2.924 | -0.6751 | Yes |
| 239 | TNRC9 |  |  | 12280 | -2.941 | -0.6744 | Yes |
| 240 | FASN |  |  | 12283 | -2.945 | -0.6726 | Yes |
| 241 | KIAA0310 |  |  | 12290 | -2.955 | -0.6710 | Yes |
| 242 | MAN2B2 |  |  | 12294 | -2.962 | -0.6692 | Yes |
| 243 | SORD |  |  | 12298 | -2.964 | -0.6674 | Yes |
| 244 | HOXB2 |  |  | 12310 | -2.977 | -0.6662 | Yes |
| 245 | CLSTN2 |  |  | 12315 | -2.985 | -0.6644 | Yes |
| 246 | C3ORF18 |  |  | 12316 | -2.986 | -0.6624 | Yes |
| 247 | DHPS |  |  | 12332 | -3.000 | -0.6615 | Yes |
| 248 | AP1M2 |  |  | 12339 | -3.008 | -0.6599 | Yes |
| 249 | BAI2 |  |  | 12346 | -3.019 | -0.6583 | Yes |
| 250 | TRIM23 |  |  | 12357 | -3.034 | -0.6570 | Yes |
| 251 | ACADSB |  |  | 12360 | -3.036 | -0.6550 | Yes |
| 252 | MIPEP |  |  | 12383 | -3.080 | -0.6546 | Yes |
| 253 | TIMP3 |  |  | 12387 | -3.085 | -0.6527 | Yes |
| 254 | ETNK2 |  |  | 12397 | -3.093 | -0.6513 | Yes |
| 255 | MAST4 |  |  | 12400 | -3.100 | -0.6493 | Yes |
| 256 | RNF43 |  |  | 12408 | -3.113 | -0.6477 | Yes |
| 257 | CD46 |  |  | 12410 | -3.115 | -0.6457 | Yes |
| 258 | NDUFA7 |  |  | 12413 | -3.121 | -0.6437 | Yes |
| 259 | MEIS3P1 |  |  | 12414 | -3.125 | -0.6415 | Yes |
| 260 | TEGT |  |  | 12420 | -3.138 | -0.6397 | Yes |
| 261 | IER3 |  |  | 12423 | -3.140 | -0.6377 | Yes |
| 262 | TFF3 |  |  | 12433 | -3.155 | -0.6363 | Yes |
| 263 | CSNK1A1 |  |  | 12437 | -3.160 | -0.6343 | Yes |
| 264 | MATN3 |  |  | 12441 | -3.165 | -0.6324 | Yes |
| 265 | TRAF5 |  |  | 12444 | -3.172 | -0.6303 | Yes |
| 266 | ACY1 |  |  | 12449 | -3.179 | -0.6285 | Yes |
| 267 | CCDC106 |  |  | 12455 | -3.190 | -0.6267 | Yes |
| 268 | LETMD1 |  |  | 12467 | -3.211 | -0.6253 | Yes |
| 269 | HHEX |  |  | 12481 | -3.239 | -0.6241 | Yes |
| 270 | RAB31 |  |  | 12488 | -3.244 | -0.6223 | Yes |
| 271 | C21ORF59 |  |  | 12497 | -3.255 | -0.6207 | Yes |
| 272 | SERPINA3 |  |  | 12498 | -3.257 | -0.6185 | Yes |
| 273 | CTDSP1 |  |  | 12499 | -3.259 | -0.6162 | Yes |
| 274 | MBTPS1 |  |  | 12512 | -3.281 | -0.6149 | Yes |
| 275 | C20ORF103 |  |  | 12514 | -3.286 | -0.6127 | Yes |
| 276 | MYO5C |  |  | 12516 | -3.289 | -0.6105 | Yes |
| 277 | HSD17B4 |  |  | 12521 | -3.294 | -0.6086 | Yes |
| 278 | PTPRT |  |  | 12525 | -3.299 | -0.6065 | Yes |
| 279 | FLJ20366 |  |  | 12529 | -3.302 | -0.6045 | Yes |
| 280 | MYB |  |  | 12539 | -3.318 | -0.6029 | Yes |
| 281 | SDC4 |  |  | 12541 | -3.322 | -0.6007 | Yes |
| 282 | IGBP1 |  |  | 12542 | -3.326 | -0.5984 | Yes |
| 283 | COL4A5 |  |  | 12545 | -3.330 | -0.5962 | Yes |
| 284 | NRIP1 |  |  | 12554 | -3.341 | -0.5946 | Yes |
| 285 | PEX12 |  |  | 12555 | -3.341 | -0.5923 | Yes |
| 286 | TPCN1 |  |  | 12559 | -3.348 | -0.5902 | Yes |
| 287 | PRLR |  |  | 12562 | -3.361 | -0.5880 | Yes |
| 288 | LASS6 |  |  | 12569 | -3.366 | -0.5862 | Yes |
| 289 | SH3BGRL |  |  | 12570 | -3.369 | -0.5839 | Yes |
| 290 | RAB27B |  |  | 12576 | -3.378 | -0.5819 | Yes |
| 291 | MYO6 |  |  | 12578 | -3.392 | -0.5797 | Yes |
| 292 | NELL2 |  |  | 12593 | -3.420 | -0.5784 | Yes |
| 293 | BCAS1 |  |  | 12594 | -3.421 | -0.5761 | Yes |
| 294 | ITGBL1 |  |  | 12600 | -3.440 | -0.5741 | Yes |
| 295 | TOB1 |  |  | 12612 | -3.453 | -0.5726 | Yes |
| 296 | GRP |  |  | 12624 | -3.476 | -0.5710 | Yes |
| 297 | BAIAP3 |  |  | 12628 | -3.484 | -0.5688 | Yes |
| 298 | ECM1 |  |  | 12630 | -3.486 | -0.5665 | Yes |
| 299 | PLEKHA6 |  |  | 12643 | -3.514 | -0.5650 | Yes |
| 300 | DACH1 |  |  | 12644 | -3.515 | -0.5626 | Yes |
| 301 | PEX19 |  |  | 12648 | -3.521 | -0.5604 | Yes |
| 302 | APBB3 |  |  | 12654 | -3.529 | -0.5584 | Yes |
| 303 | AP2B1 |  |  | 12670 | -3.557 | -0.5571 | Yes |
| 304 | HDAC11 |  |  | 12678 | -3.574 | -0.5552 | Yes |
| 305 | HIST1H2BD |  |  | 12685 | -3.585 | -0.5532 | Yes |
| 306 | PREPL |  |  | 12686 | -3.588 | -0.5507 | Yes |
| 307 | MKNK2 |  |  | 12691 | -3.600 | -0.5485 | Yes |
| 308 | KIAA0423 |  |  | 12692 | -3.603 | -0.5461 | Yes |
| 309 | ELOVL2 |  |  | 12695 | -3.604 | -0.5437 | Yes |
| 310 | CA12 |  |  | 12705 | -3.627 | -0.5419 | Yes |
| 311 | GALNT7 |  |  | 12708 | -3.629 | -0.5396 | Yes |
| 312 | LRPAP1 |  |  | 12709 | -3.635 | -0.5371 | Yes |
| 313 | TUSC2 |  |  | 12728 | -3.684 | -0.5360 | Yes |
| 314 | RAI17 |  |  | 12730 | -3.686 | -0.5335 | Yes |
| 315 | TGFB3 |  |  | 12734 | -3.702 | -0.5312 | Yes |
| 316 | CYP2B7P1 |  |  | 12735 | -3.703 | -0.5286 | Yes |
| 317 | C5ORF5 |  |  | 12738 | -3.711 | -0.5262 | Yes |
| 318 | C9ORF7 |  |  | 12763 | -3.780 | -0.5255 | Yes |
| 319 | FLNB |  |  | 12764 | -3.781 | -0.5229 | Yes |
| 320 | ALCAM |  |  | 12765 | -3.783 | -0.5203 | Yes |
| 321 | GLUD1 |  |  | 12774 | -3.804 | -0.5183 | Yes |
| 322 | TFF1 |  |  | 12775 | -3.809 | -0.5157 | Yes |
| 323 | SALL2 |  |  | 12781 | -3.823 | -0.5134 | Yes |
| 324 | ABCD3 |  |  | 12788 | -3.840 | -0.5112 | Yes |
| 325 | COMP |  |  | 12792 | -3.867 | -0.5088 | Yes |
| 326 | TBX3 |  |  | 12795 | -3.880 | -0.5063 | Yes |
| 327 | C10ORF116 |  |  | 12803 | -3.895 | -0.5042 | Yes |
| 328 | COASY |  |  | 12810 | -3.911 | -0.5019 | Yes |
| 329 | CTSF |  |  | 12816 | -3.921 | -0.4996 | Yes |
| 330 | PCMTD2 |  |  | 12818 | -3.925 | -0.4970 | Yes |
| 331 | COX6C |  |  | 12819 | -3.930 | -0.4943 | Yes |
| 332 | RNF103 |  |  | 12823 | -3.938 | -0.4918 | Yes |
| 333 | CHMP2A |  |  | 12827 | -3.946 | -0.4893 | Yes |
| 334 | SEPP1 |  |  | 12828 | -3.949 | -0.4866 | Yes |
| 335 | MTL5 |  |  | 12830 | -3.952 | -0.4840 | Yes |
| 336 | GRPR |  |  | 12837 | -3.971 | -0.4817 | Yes |
| 337 | NPDC1 |  |  | 12839 | -3.972 | -0.4790 | Yes |
| 338 | CAP2 |  |  | 12840 | -3.972 | -0.4763 | Yes |
| 339 | PLA2G10 |  |  | 12843 | -3.988 | -0.4737 | Yes |
| 340 | FBP1 |  |  | 12849 | -4.003 | -0.4714 | Yes |
| 341 | SLC27A2 |  |  | 12852 | -4.014 | -0.4687 | Yes |
| 342 | SORL1 |  |  | 12857 | -4.023 | -0.4663 | Yes |
| 343 | HIST1H2AC |  |  | 12865 | -4.048 | -0.4640 | Yes |
| 344 | PDCD4 |  |  | 12873 | -4.072 | -0.4618 | Yes |
| 345 | FAM102A |  |  | 12880 | -4.089 | -0.4594 | Yes |
| 346 | SPARCL1 |  |  | 12889 | -4.108 | -0.4572 | Yes |
| 347 | FAAH |  |  | 12893 | -4.137 | -0.4546 | Yes |
| 348 | NBEA |  |  | 12894 | -4.140 | -0.4518 | Yes |
| 349 | MRPS30 |  |  | 12895 | -4.143 | -0.4489 | Yes |
| 350 | IGFBP2 |  |  | 12897 | -4.147 | -0.4461 | Yes |
| 351 | LAMA5 |  |  | 12907 | -4.167 | -0.4440 | Yes |
| 352 | BMPR1B |  |  | 12909 | -4.170 | -0.4412 | Yes |
| 353 | ERBB2IP |  |  | 12910 | -4.180 | -0.4383 | Yes |
| 354 | PIGH |  |  | 12912 | -4.186 | -0.4355 | Yes |
| 355 | ITGB5 |  |  | 12921 | -4.213 | -0.4332 | Yes |
| 356 | HAGH |  |  | 12925 | -4.250 | -0.4305 | Yes |
| 357 | TMEM16A |  |  | 12927 | -4.253 | -0.4277 | Yes |
| 358 | CXORF40B |  |  | 12928 | -4.271 | -0.4247 | Yes |
| 359 | PNMA1 |  |  | 12929 | -4.272 | -0.4218 | Yes |
| 360 | AGR2 |  |  | 12930 | -4.272 | -0.4188 | Yes |
| 361 | STARD13 |  |  | 12931 | -4.273 | -0.4159 | Yes |
| 362 | C5ORF15 |  |  | 12937 | -4.282 | -0.4133 | Yes |
| 363 | CNNM4 |  |  | 12938 | -4.283 | -0.4104 | Yes |
| 364 | UQCRQ |  |  | 12939 | -4.284 | -0.4074 | Yes |
| 365 | C5ORF21 |  |  | 12941 | -4.293 | -0.4046 | Yes |
| 366 | FARP2 |  |  | 12943 | -4.310 | -0.4017 | Yes |
| 367 | LZTFL1 |  |  | 12950 | -4.317 | -0.3992 | Yes |
| 368 | STK39 |  |  | 12951 | -4.321 | -0.3962 | Yes |
| 369 | SLC7A8 |  |  | 12952 | -4.333 | -0.3932 | Yes |
| 370 | TINP1 |  |  | 12956 | -4.342 | -0.3904 | Yes |
| 371 | LAMB2 |  |  | 12959 | -4.349 | -0.3876 | Yes |
| 372 | TSPAN1 |  |  | 12960 | -4.359 | -0.3846 | Yes |
| 373 | MRPS27 |  |  | 12964 | -4.371 | -0.3818 | Yes |
| 374 | TMEM66 |  |  | 12974 | -4.400 | -0.3795 | Yes |
| 375 | CROT |  |  | 12986 | -4.423 | -0.3773 | Yes |
| 376 | ASPN |  |  | 12993 | -4.438 | -0.3747 | Yes |
| 377 | PMVK |  |  | 12998 | -4.449 | -0.3720 | Yes |
| 378 | DUSP4 |  |  | 13001 | -4.456 | -0.3690 | Yes |
| 379 | GATA3 |  |  | 13014 | -4.509 | -0.3669 | Yes |
| 380 | GFRA1 |  |  | 13018 | -4.523 | -0.3640 | Yes |
| 381 | HSPB1 |  |  | 13020 | -4.526 | -0.3610 | Yes |
| 382 | ABCA3 |  |  | 13025 | -4.531 | -0.3581 | Yes |
| 383 | ZNF91 |  |  | 13035 | -4.577 | -0.3557 | Yes |
| 384 | BTG2 |  |  | 13039 | -4.613 | -0.3528 | Yes |
| 385 | HTRA1 |  |  | 13042 | -4.623 | -0.3497 | Yes |
| 386 | SLC9A3R1 |  |  | 13043 | -4.624 | -0.3465 | Yes |
| 387 | SUCLG2 |  |  | 13046 | -4.630 | -0.3435 | Yes |
| 388 | PALM |  |  | 13051 | -4.654 | -0.3406 | Yes |
| 389 | FDXR |  |  | 13054 | -4.663 | -0.3376 | Yes |
| 390 | DHCR24 |  |  | 13061 | -4.682 | -0.3348 | Yes |
| 391 | RASA1 |  |  | 13063 | -4.690 | -0.3316 | Yes |
| 392 | DAK |  |  | 13064 | -4.690 | -0.3284 | Yes |
| 393 | ABAT |  |  | 13071 | -4.721 | -0.3256 | Yes |
| 394 | SLC4A8 |  |  | 13080 | -4.751 | -0.3230 | Yes |
| 395 | KIF3B |  |  | 13084 | -4.771 | -0.3199 | Yes |
| 396 | TENC1 |  |  | 13098 | -4.809 | -0.3176 | Yes |
| 397 | FOXA1 |  |  | 13107 | -4.857 | -0.3149 | Yes |
| 398 | IMPDH2 |  |  | 13108 | -4.860 | -0.3116 | Yes |
| 399 | SIAH2 |  |  | 13112 | -4.869 | -0.3084 | Yes |
| 400 | TSPAN13 |  |  | 13115 | -4.889 | -0.3052 | Yes |
| 401 | SCGB1D2 |  |  | 13121 | -4.913 | -0.3022 | Yes |
| 402 | PIB5PA |  |  | 13125 | -4.921 | -0.2991 | Yes |
| 403 | NAT1 |  |  | 13126 | -4.922 | -0.2957 | Yes |
| 404 | PLAT |  |  | 13127 | -4.926 | -0.2923 | Yes |
| 405 | HEBP1 |  |  | 13134 | -4.962 | -0.2894 | Yes |
| 406 | AKR7A2 |  |  | 13140 | -4.985 | -0.2863 | Yes |
| 407 | MCCC2 |  |  | 13143 | -5.005 | -0.2830 | Yes |
| 408 | DSU |  |  | 13144 | -5.008 | -0.2796 | Yes |
| 409 | CAMK2N1 |  |  | 13145 | -5.010 | -0.2761 | Yes |
| 410 | PPT1 |  |  | 13146 | -5.015 | -0.2727 | Yes |
| 411 | SLC39A6 |  |  | 13151 | -5.039 | -0.2695 | Yes |
| 412 | PVRL2 |  |  | 13152 | -5.049 | -0.2660 | Yes |
| 413 | CCNG2 |  |  | 13157 | -5.088 | -0.2628 | Yes |
| 414 | RARRES3 |  |  | 13162 | -5.126 | -0.2596 | Yes |
| 415 | C20ORF23 |  |  | 13167 | -5.158 | -0.2564 | Yes |
| 416 | KIAA0232 |  |  | 13171 | -5.183 | -0.2530 | Yes |
| 417 | TRPS1 |  |  | 13172 | -5.184 | -0.2495 | Yes |
| 418 | THBS4 |  |  | 13174 | -5.197 | -0.2460 | Yes |
| 419 | HRASLS3 |  |  | 13176 | -5.210 | -0.2425 | Yes |
| 420 | DCI |  |  | 13177 | -5.217 | -0.2389 | Yes |
| 421 | PH-4 |  |  | 13179 | -5.236 | -0.2353 | Yes |
| 422 | CRIP2 |  |  | 13180 | -5.244 | -0.2317 | Yes |
| 423 | SMA3 |  |  | 13185 | -5.279 | -0.2284 | Yes |
| 424 | GALNAC4S-6ST |  |  | 13192 | -5.342 | -0.2252 | Yes |
| 425 | C1ORF78 |  |  | 13194 | -5.346 | -0.2216 | Yes |
| 426 | FUT8 |  |  | 13195 | -5.349 | -0.2179 | Yes |
| 427 | PECI |  |  | 13201 | -5.385 | -0.2146 | Yes |
| 428 | CIRBP |  |  | 13206 | -5.436 | -0.2112 | Yes |
| 429 | SYNJ2BP |  |  | 13207 | -5.444 | -0.2074 | Yes |
| 430 | WFS1 |  |  | 13210 | -5.457 | -0.2038 | Yes |
| 431 | FAH |  |  | 13211 | -5.503 | -0.2000 | Yes |
| 432 | HHAT |  |  | 13212 | -5.503 | -0.1962 | Yes |
| 433 | GLI3 |  |  | 13213 | -5.527 | -0.1924 | Yes |
| 434 | CYB5R1 |  |  | 13214 | -5.529 | -0.1886 | Yes |
| 435 | ABLIM3 |  |  | 13222 | -5.596 | -0.1853 | Yes |
| 436 | AR |  |  | 13224 | -5.617 | -0.1815 | Yes |
| 437 | TBC1D9 |  |  | 13226 | -5.639 | -0.1777 | Yes |
| 438 | IGFBP4 |  |  | 13227 | -5.654 | -0.1738 | Yes |
| 439 | EEF1A2 |  |  | 13232 | -5.711 | -0.1702 | Yes |
| 440 | DIP13B |  |  | 13233 | -5.716 | -0.1662 | Yes |
| 441 | LRBA |  |  | 13234 | -5.717 | -0.1623 | Yes |
| 442 | SCUBE2 |  |  | 13239 | -5.755 | -0.1587 | Yes |
| 443 | C10ORF56 |  |  | 13240 | -5.756 | -0.1547 | Yes |
| 444 | PGR |  |  | 13241 | -5.770 | -0.1507 | Yes |
| 445 | PDZK1 |  |  | 13242 | -5.783 | -0.1467 | Yes |
| 446 | GSTM3 |  |  | 13252 | -5.994 | -0.1433 | Yes |
| 447 | KCTD3 |  |  | 13254 | -6.009 | -0.1393 | Yes |
| 448 | HSPB8 |  |  | 13256 | -6.029 | -0.1352 | Yes |
| 449 | DNALI1 |  |  | 13258 | -6.058 | -0.1311 | Yes |
| 450 | TPBG |  |  | 13260 | -6.071 | -0.1270 | Yes |
| 451 | TMBIM4 |  |  | 13262 | -6.100 | -0.1229 | Yes |
| 452 | REPS2 |  |  | 13263 | -6.105 | -0.1187 | Yes |
| 453 | PIGT |  |  | 13264 | -6.118 | -0.1144 | Yes |
| 454 | VAV3 |  |  | 13265 | -6.141 | -0.1102 | Yes |
| 455 | UGDH |  |  | 13266 | -6.157 | -0.1060 | Yes |
| 456 | BHLHB2 |  |  | 13270 | -6.208 | -0.1019 | Yes |
| 457 | FLJ20152 |  |  | 13272 | -6.223 | -0.0977 | Yes |
| 458 | EMP2 |  |  | 13273 | -6.228 | -0.0934 | Yes |
| 459 | IRX5 |  |  | 13278 | -6.313 | -0.0894 | Yes |
| 460 | DNAJC12 |  |  | 13287 | -6.382 | -0.0856 | Yes |
| 461 | LASS2 |  |  | 13288 | -6.409 | -0.0812 | Yes |
| 462 | FAM63A |  |  | 13291 | -6.442 | -0.0769 | Yes |
| 463 | SCCPDH |  |  | 13292 | -6.455 | -0.0725 | Yes |
| 464 | LRRC17 |  |  | 13294 | -6.494 | -0.0681 | Yes |
| 465 | UGCG |  |  | 13297 | -6.552 | -0.0637 | Yes |
| 466 | SLC16A6 |  |  | 13298 | -6.552 | -0.0592 | Yes |
| 467 | GPD1L |  |  | 13299 | -6.602 | -0.0547 | Yes |
| 468 | TCEAL1 |  |  | 13302 | -6.686 | -0.0502 | Yes |
| 469 | RGL2 |  |  | 13304 | -6.810 | -0.0456 | Yes |
| 470 | KLHDC2 |  |  | 13305 | -6.820 | -0.0409 | Yes |
| 471 | KIAA0040 |  |  | 13309 | -7.057 | -0.0363 | Yes |
| 472 | XBP1 |  |  | 13311 | -7.068 | -0.0315 | Yes |
| 473 | IRS1 |  |  | 13313 | -7.188 | -0.0266 | Yes |
| 474 | LRP10 |  |  | 13314 | -7.445 | -0.0215 | Yes |
| 475 | SSH3 |  |  | 13315 | -7.460 | -0.0164 | Yes |
| 476 | FLJ20273 |  |  | 13316 | -7.530 | -0.0112 | Yes |
| 477 | KRT18 |  |  | 13317 | -7.649 | -0.0059 | Yes |
| 478 | ANXA9 |  |  | 13320 | -8.796 | -0.0000 | Yes |
Table: GSEA details [plain text format]

  

Fig 2: BRCA\_ER\_POS: Random ES distribution      
 Gene set null distribution of ES for **BRCA\_ER\_POS**
